# Comparative Genetic Diversity and Population Structure of Wild *Atalantia* from Taiwan and Sri Lanka Using SSR Markers

**DOI:** 10.3390/plants15040570

**Published:** 2026-02-11

**Authors:** Piumi Chathurika Palangasinghe, Huie-Chuan Shih, Yi-Han Chang, Wasantha Kumara Liyanage, Annamalai Muthusamy, Meng-Shin Shiao, Yu-Chung Chiang

**Affiliations:** 1 Department of Biological Sciences, National Sun Yat-sen University, 70 Lienhai Road, Kaohsiung 80424, Taiwan; piumipalangasinghe@gmail.com; 2 Department of Nursing, Meiho University, Pingtung 91202, Taiwan; x00002213@meiho.edu.tw; 3 Hengchun Research Center, Taiwan Forestry Research Institute, Pingtung 94644, Taiwan; changii0331@tfri.gov.tw; 4 Department of Agricultural Biology, Faculty of Agriculture, University of Ruhuna, Mapalana 81100, Sri Lanka; wasantha@agri.ruh.ac.lk; 5 Department of Plant Sciences, Manipal School of Life Sciences, Manipal Academy of Higher Education (MAHE), Manipal 576104, Karnataka, India; a.msamy@manipal.edu; 6 Research Laboratory Section, Offices of Health Science Research, Faculty of Medicine Ramathibodi Hospital, Mahidol University, Bangkok 10400, Thailand; 7 Department of Biomedical Science and Environment Biology, Kaohsiung Medical University, Kaohsiung 80708, Taiwan

**Keywords:** genetic diversity, population structure, *Atalantia*, microsatellites, conservation, habitat fragmentation

## Abstract

Understanding genetic diversity and population structure in wild Citrus relatives is crucial for conservation and crop improvement. Here, we examined genetic variation in *Atalantia buxifolia* from the island of Taiwan and *Atalantia ceylanica* from Sri Lanka using 21 transferable microsatellite (SSR) markers originally developed for *Citrus*. A total of 132 individuals from 13 populations were genotyped. Both species exhibited moderate levels of polymorphism, with *A. buxifolia* showing slightly higher allelic richness and heterozygosity than *A. ceylanica*. Analysis of molecular variance indicated that most genetic variation occurred within individuals (68% in *A. buxifolia* and 82% in *A. ceylanica*), while moderate population differentiation was detected (*F_ST_* = 0.356 and 0.204, respectively). STRUCTURE, DAPC, PCoA, and *F_ST_* analyses revealed distinct regional clustering in *A. buxifolia*, particularly in the Shoushan population, whereas populations of *A. ceylanica* were weakly structured. Monmonier’s analysis identified genetic barriers only in *A. buxifolia*, and BayesAss indicated high self-recruitment and localized gene flow in both species. Overall, these results suggest high within-population genetic diversity but limited connectivity among populations, shaped by geographic isolation and habitat fragmentation. Our findings provide a baseline for conservation planning in *Atalantia* populations and highlight the importance of maintaining habitat connectivity to preserve genetic resilience.

## 1. Introduction

The genus *Atalantia* (Rutaceae) is commonly found in tropical Asia and comprises small trees and shrubs [1]. It includes several narrowly defined species and can be characterized by glandular leaves, fragrant white flowers, and berry fruits [1]. *Atalantia* is closely related to cultivated *Citrus* species and other members of the subfamily Aurantioideae, yet it forms a distinct evolutionary lineage within the Rutaceae family [2,3,4,5]. The genus plays an important ecological role by providing fruits for frugivorous animals and serves as a genetic reservoir for traits with potential agricultural and horticultural importance [6]. Preserving genetic variation within *Atalantia* species is crucial for maintaining their adaptive potential and resilience to environmental change [7].

The two species studied here, *Atalantia ceylanica* (Arn.) Oliv. and *Atalantia buxifolia* (Poir.) Oliv. ex Benth., have distinct geographic ranges and biological characteristics. *A. ceylanica*, native to Sri Lanka and southern India, grows as a densely branched, spiny shrub with ovate to elliptic leaves measuring 4–12 cm long and fruits about 1–1.5 cm in diameter [1]. In contrast, *A. buxifolia*, distributed across southern China and the island of Taiwan, forms a shrub with smaller spiny leaves and produces small black berry-type fruits [2,3]. Both species have limited distributions, often restricted to isolated forest fragments [2]. Such isolation may enhance genetic differentiation among populations and lead to distinct evolutionary paths [5]. Populations are subject to selective pressures from variations in climate, soil, and biotic interactions, and ongoing habitat degradation and human activities pose additional threats [2,5]. Understanding genetic variation within and between these species can guide conservation strategies and identify evolutionarily significant units for protection [7].

Studies based on molecular markers have been the primary tool for investigating genetic diversity in Rutaceae; however, the genetic diversity and population structure of *Atalantia* species remain poorly understood. Isozyme studies placed *A. ceylanica* with the “lime-lemon-citron” group of *Citrus*, along with species from the Australian genus *Microcitrus*, suggesting a complex evolutionary relationship within Aurantioideae [4]. Randomly Amplified Polymorphic DNA (RAPD) and Inter Simple Sequence Repeats (ISSR) on species from the Western Ghats, such as *A. racemosa*, *A. wightii*, and *A. monophylla*, revealed more genetic variation within than between species [2]. These findings suggest that current taxonomic classifications may misjudge the gene flow and hybridization within the genus. Similarly, a RAPD-based study on *A. ceylanica* highlighted its significant genetic divergence from all tested *Citrus* accessions, highlighting its genetic distinctiveness [8]. Comparative studies across related genera provide additional context for interpreting genetic variations in *Atalantia*.

Comparing to isozymes and dominant markers such as RAPD, codominant microsatellite markers (Simple Sequence Repeats—SSRs), offer higher resolution and allow precise estimation of allele frequencies, heterozygosity, and population structure [9]. SSRs are multiallelic, widely distributed across the genome, and often transferable across closely related species, making them highly suitable for non-model taxa like *Atalantia* [10,11]. In *Citrus* and relatives, SSR markers have effectively outlined both inter- and intraspecific variation, identifying major taxonomic groups and ancestral lineages, including citron, pummelo, and mandarin [12,13]. Microsatellite analyses in *Citrus* germplasm demonstrate that closely related genera can show varying levels of interspecific divergence. For example, kumquats (*Fortunella*) cluster within *Citrus*, whereas *Poncirus trifoliata* occupies a basal position relative to cultivated *Citrus* species [13]. Despite these advantages, no published studies have used codominant SSR markers specifically for *Atalantia*, leaving a significant gap in our understanding of genetic variation and population connectivity within the genus.

The fact that both species are found on large continental islands that share broadly comparable geological histories provides an opportunity to examine patterns of genetic divergence in insular lineages under different ecological contexts. Comparative population genetic studies of these species are still lacking. Although *A. buxifolia* and *A. ceylanica* are closely related species, they occupy distinct ecological and geographical ranges that justify separate genetic analyses. *A. buxifolia* is found in coastal and secondary forests across subtropical East and Southeast Asia [14], while *A. ceylanica* is in tropical evergreen and dry zone forests endemic to Sri Lanka and southern India [15]. Moreover, morphological divergence in leaf structure, fruit coloration, and reproductive traits indicates independent evolutionary paths [16].

Developing on these observations, the present study focuses on genetic differentiation between and within *A. ceylanica* and *A. buxifolia*, representing disjunct island and mainland lineages in Sri Lanka and East Asia, respectively. Using microsatellite markers originally developed for *Citrus*, we aim to quantify intra specific genetic variation, assess population structure, and compare genetic diversity metrics between these species. By analyzing genetic variation in these geographically disjunct taxa, this study aims to explore how isolation and habitat conditions have shaped population structure and evolutionary trajectories and to provide insights into historical connectivity, potential gene flow, and conservation priorities for *Atalantia* species across tropical Asia.

## 2. Results

### 2.1. Polymorphism Analysis Based on Microsatellite Markers

Genetic diversity indices, number of alleles (*A*), number of effective alleles (*A_e_*), observed heterozygosity (*H_o_*), and expected heterozygosity (*H_e_*) were calculated for the 21 microsatellite loci (Tables S2 and S3). In *Atalantia buxifolia*, the Shoushan (SH) population showed the moderate effective number of alleles (*A_e_*) at most of the loci, and the highest number of *A_e_* was observed for Cit_24, Cit_25 and Cit_31. For AL population, the highest *A_e_* was observed at loci Cit_20, Cit_24, Cit_29 and Cit_34. Likewise, the Longchi (LO) population also showed similar diversity at Cit_26. The Mituo (MI) population, in terms of allelic diversity, at Cit_26 showed the highest effective number of alleles. In Neipu (NP), the only cultivated population, showed the highest *A_e_* at Cit_24 and Cit_26. The Kenting (KE) population showed relatively high allelic diversity at Cit_7, Cit_10, Cit_19, Cit_24 and Cit_26. In Dashanmu (DA), Cit_10, Cit_24 and Cit_29 showed the highest diversity. In Chiniuiling (CH), Cit_2 and Cit_24 showed the highest diversity. In the Guishan (GU) population, loci Cit_2, Cit_20, and Cit_24 showed the highest diversity. Overall, Cit_10 and Cit_24 were the loci with high polymorphism across populations, while Cit_3, Cit_4, and Cit_9 were monomorphic in all *A. buxifolia* populations.

In *Atalantia ceylanica*, relatively less polymorphism was observed per locus. In the Palatuwa (PL) population, the highest diversity was observed at Cit_29. In the Attudawa (AT) population, loci Cit_24 and Cit_29 showed the highest variation. In Malimboda (MA), the population at Cit_10 and Cit_24 showed the highest allelic variation. In the Morawaka (MO) population, Cit_10, Cit _24 and Cit_31 showed the highest variation. Loci Cit_10 and Cit_24 were (*H_o_* up to 1.00) polymorphic and heterozygous across all populations, while some loci (Cit_3, Cit_4, Cit_8, Cit_14, Cit_15, Cit_23, Cit_30, Cit_34) were completely lacking variation in at least two populations. The most polymorphic loci, Cit_9, Cit_10, Cit_18, Cit_19, Cit_24, Cit_26, Cit_29, and Cit_31, were the sites of significant deviations (*p* < 0.05) from Hardy–Weinberg equilibrium. Overall, genetic diversity varied among populations, with Cit_10 and Cit_24 showing the highest polymorphism across both species, while several loci were monomorphic (Tables S2 and S3).

### 2.2. Genetic Diversity and Structure of the Two Species

The AMOVA revealed that 32% of the total genetic variation occurred among populations and 68% within individuals of *A. buxifolia* (Table 1). The overall fixation index was high (*F_ST_* = 0.356, *p* < 0.05). The degree of inbreeding (*F_IS_*) was −0.189 and the overall inbreeding (*F_IT_*) was 0.234, although the *F_IS_* was not statistically significant. For *A. ceylanica* populations, AMOVA indicated that 18% of the total genetic variation occurred among populations, whereas 82% of variation was within individuals (Table 2). The overall fixation index was high, similarly to *A. buxifolia* (*F_ST_* = 0.204, *p* < 0.05). The degree of inbreeding (*F_IS_*) and the overall inbreeding (*F_IT_*) were statistically not significant, with values of −0.207 and 0.039.

**Figure 1:**
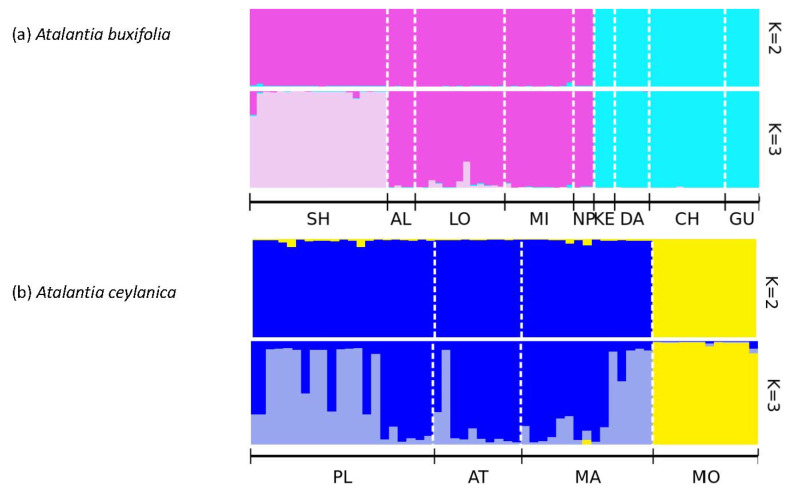
Bayesian clustering analysis of genetic structure in (**a**) *Atalantia buxifolia* and (**b**) *A. ceylanica* populations based on microsatellite loci using STRUCTURE. Each vertical bar represents an individual, and colors indicate inferred genetic clusters at K = 2 and K = 3. Population codes correspond to sampling locations listed in Table 1.

Bayesian clustering analysis in STRUCTURE identified K = 2 as the statistically optimal number of clusters for both *A. buxifolia* and *A. ceylanica*. Although K = 3 is not the most optimal solution statistically, it provided the more biologically meaningful interpretation for both *A. buxifolia* and *A. ceylanica* (Figure 1, Figures S1 and S2, Tables S4 and S5). For *A. buxifolia*, populations SH, AL, LO, MI, and NP were grouped into one cluster, whereas KE, DA, CH, and GU formed a second distinct cluster. Notably, NP (from Pingtung) was genetically closer to Kaohsiung populations despite its geographic separation. At K = 3, AL, LO, MI, and NP formed a separate cluster, while SH appeared distinct. Similarly, in *A. ceylanica*, populations PL, AT, and MA clustered together and showed evidence of gene flow, whereas MO formed a separate group.

DAPC was performed to characterize the genetic structure among individuals of *A. buxifolia* and *A. ceylanica*. Linear Discriminants 1 and 2 (LD1 and LD2) accounted for 91.19% of cumulative variance for *A. buxifolia*, while LD1, LD2 and LD3 together accounted for 99.99% cumulative variance for *A. ceylanica* (Figure S3A,B). For *A. buxifolia*, the LD3 was not included, as the first two linear discriminants showed enough variation.

**Figure 2:**
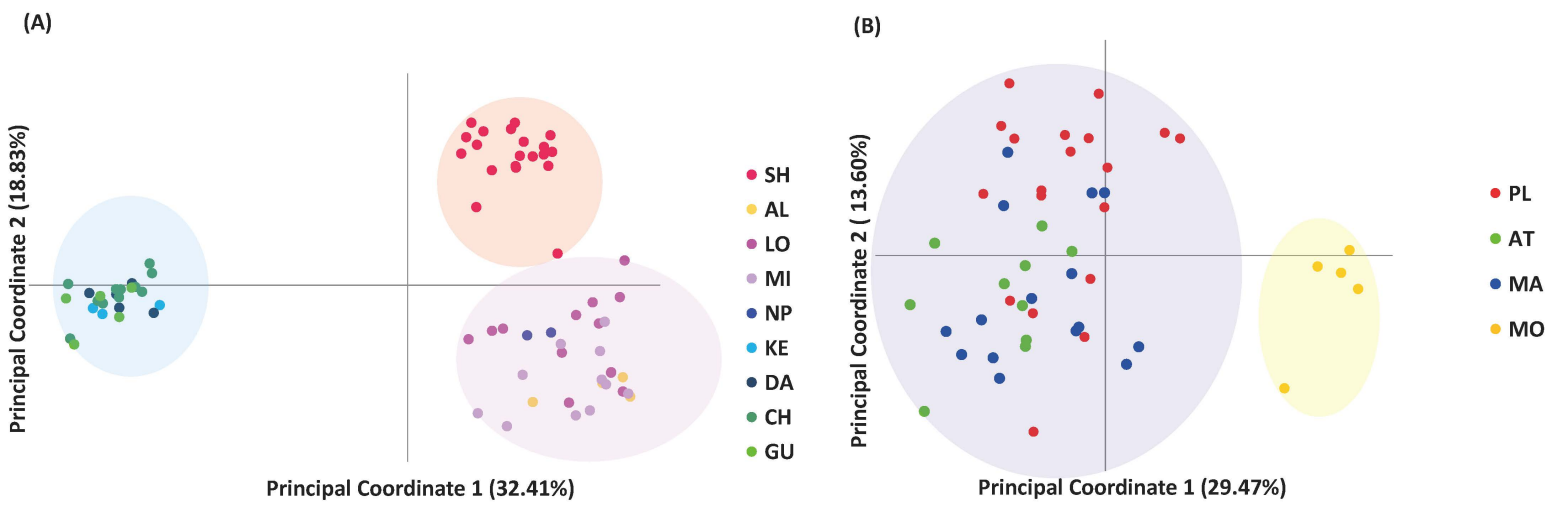
Principal Coordinate Analysis (PCoA) based on 21 microsatellite loci. (**A**) PCoA of *Atalantia buxifolia* populations sampled across the southern part of the island of Taiwan. Each point represents an individual, and colors indicate different populations. The first two principal coordinates explain 32.41% and 18.83% of the total genetic variation, respectively. (**B**) PCoA of *Atalantia ceylanica* populations from Sri Lanka. Each point represents an individual, and colors correspond to populations Palatuwa (PL), Attudawa (AT), Malimboda (MA), and Morawaka (MO). The first two principal coordinates explain 29.47% and 13.60% of the total genetic variation, respectively.

The principal coordinate analysis (PCoA) was able to identify three distinct clusters for *A. buxifolia* complying with the STRUCTURE results separating Pingtung and Kaohsiung populations and separating the SH population on PC2 (Figure 2A). The first and second axes explained 32.41% and 18.83% of the total genetic variation for *A. buxifolia*. For *A.* *ceylanica*, PCoA revealed a wide dispersion of individuals with overlapping clusters among the three populations (Figure 2B). However, the MO population was separated along the first axis. The first and second axes explained 29.47% and 13.60% of the total genetic variation, respectively.

### 2.3. Genetic Barriers Were Detected Between Different Geographic Regions

**Figure 3:**
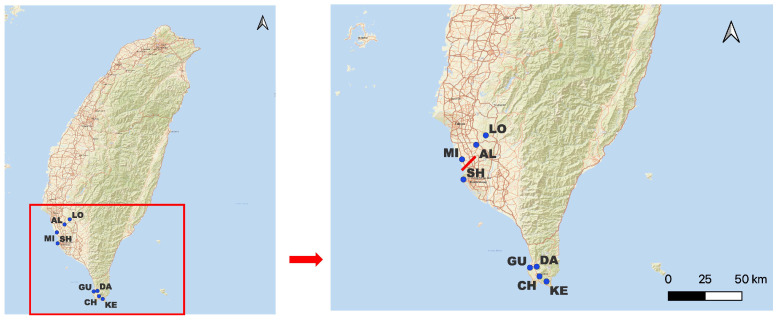
Genetic barriers among *Atalantia buxifolia* populations inferred using Monmonier’s algorithm based on pairwise genetic distances from 21 microsatellite loci. Blue points represent sampling locations, and population codes indicate individual populations. The red solid line indicates the genetic barrier, separating populations. Note that the NP populat The red solid line indicates the genetic barrier, separating populations.

Significant genetic barriers among *A. buxifolia* populations were detected using Monmonier’s algorithm [17]. Barrier paths (Figure 3) indicate clear separation of the SH population from other populations in the Kaohsiung region as shown in the previous analysis. In contrast, populations KE, DA, CH, and GU showed no detectable barriers, suggesting higher genetic connectivity within this area. The NP population, derived from a cultivated orchard, was excluded to avoid confusing natural genetic patterns.

**Figure 4:**
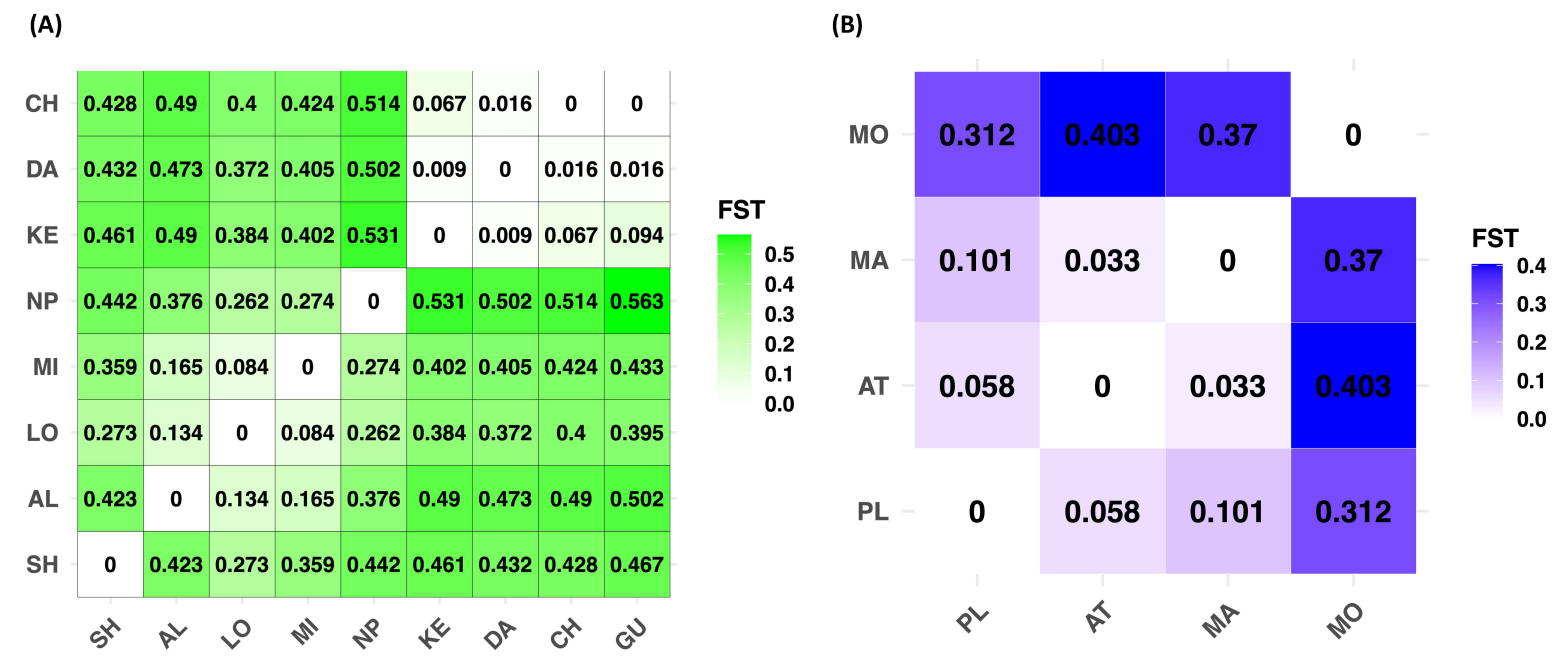
Heatmaps displaying pairwise population F*_ST_* values based on microsatellite data for (**A**) *A. buxifolia* and (**B**) *A. ceylanica*.

Estimates of pairwise population genetic differentiation varied among the nine populations of *A. buxifolia* (Figure 4A), with *F_ST_* values ranging from zero (e.g., CH vs. GU = 0.000; DA vs. KE = 0.009) to high differentiation between geographically and genetically isolated populations (e.g., AL vs. KE = 0.490; NP vs. KE = 0.531). Populations DA, CH, GU, and KE exhibited consistently low pairwise *F_ST_* values among themselves, indicating substantial gene flow and weak genetic differentiation within this group. In contrast, SH, AL, and NP showed higher *F_ST_* values relative to most other populations, suggesting that these populations are more genetically differentiated from the remainder of the species range. Intermediate levels of differentiation were observed for LO and MI, which displayed moderate *F_ST_* values when compared with both the highly differentiated populations (SH, AL, NP) and the less differentiated group (DA, CH, GU, KE).

For *A. ceylanica*, pairwise population genetic differentiation among the four populations was variable. *F_ST_* values were low between PL, AT, and MA (0.033–0.101). In contrast, MO was highly differentiated from the other populations (*F_ST_* = 0.312–0.403) (Figure 4B).

### 2.4. Stronger Self-Recruitment Was Detected in All Populations of A. buxifolia than Those of A. ceylanica

The previous analyses, i.e., STRUCTURE and PCoA analyses, indicated that populations in the Pingtung area were genetically very similar, exhibiting low differentiation. Therefore, these populations were combined into a single unit (PI) for subsequent analyses, allowing clearer interpretation of genetic structure and diversity across regions. The analysis by BayesAss (version 3.0.5.6) revealed consistently high levels of self-recruitment (i.e., the proportion of individuals originating from the same population, represented by the value along the diagonal) across most populations, with values ranging from 68.89% in MI to 95.40% in PI populations (Table 3). The most significant migration events were observed in LO populations, which received 24.43% and 18.38% gene flow from MI and AL populations, respectively. All remaining migration estimates were below 5%.

Self-recruitment rates varied among *A. ceylanica* populations, ranging from 68% in PL to 94% in MO (Table 4). Overall, *A. ceylanica* showed a lower self-recruitment rate than *A. buxifolia*.

## 3. Discussion

This study, based on microsatellite markers of *Atalantia buxifolia* from the island of Taiwan and *Atalantia ceylanica* from Sri Lanka, indicates modest genetic variation within each population. Due to their codominant nature and reproducibility across species, microsatellites have become a popular tool in plant population genetics [11]. Recently, microsatellites have been used for wild and cultivated species, including members of the Rutaceae family [18,19]. Due to their reproducibility, microsatellites originally developed for *Citrus* species [20] were effective in genotyping *Atalantia* populations, highlighting the broad applicability of microsatellite markers for assessing genetic diversity in non-model plants [21].

The mean number of observed alleles per locus (*A*) was low in both species, indicating limited allelic richness. *A. buxifolia* showed slightly higher richness (mean *A* = 1.962) than *A. ceylanica* (mean *A* = 1.738), suggesting a relatively more diverse gene pool. These values are lower than previous reports of 7–12 alleles per locus in *A. buxifolia* and *A. monophyla* [2,22], likely due to smaller, fragmented populations in this study and possible cross-species microsatellite limitations. Alternatively, the reduced allelic richness may reflect biological influences such as historical bottlenecks, inbreeding, or limited gene flow [23]. The effective number of alleles (*A_e_*), which considers allele frequencies, showed a similar pattern: in *A. buxifolia*, *N_e_* ranged from 1.32 to 1.43, indicating moderate diversity, consistent with findings in other *Atalantia* species [2]. In *A. ceylanica*, *A_e_* ranged from 1.17 to 1.34, reflecting the trend in *A. buxifolia*.

Observed heterozygosity (*H_o_*) was slightly higher than expected heterozygosity (*H_e_*) in both *A. buxifolia* (*H_o_* = 0.373; *H_e_* = 0.297) and *A. ceylanica* (*H_o_* = 0.272; *H_e_* = 0.216), indicating an excess of heterozygotes. Although our dataset shows an apparent excess of heterozygosity (*H_o_* > *H_e_*), patterns like this are not common in natural plant populations. Therefore, this deviation is more likely due to methodological or sampling factors rather than a true biological signal. Potential contributors include small sample sizes, inadvertent sampling of substructured populations (Wahlund effect), increased outcrossing, or the presence of null alleles, which are common when using cross-species SSR markers. However, an excess of heterozygotes has been reported in previous studies in the Rutaceae family, such as in *Citrus hongheensis* Y.Ye, X.Liu, S.Q.Ding & M.Liang (*H*_o_ = 0.5195; *H_e_* = 0.3520) [24]. Cross-species SSR amplification increases the likelihood of null alleles, which may inflate heterozygosity or cause deviations from the Hardy–Weinberg equilibrium. Compared with related species, such as *Citrus × sinensis* (L.) Osbeck (*H_o_* = 0.50–0.70) [13] and wild *Zanthoxylum nitidum* (Roxb.) DC. (*H_o_* > 0.60) [25], heterozygosity in *Atalantia* populations was relatively low, likely due to restricted geographic range, habitat fragmentation, and anthropogenic pressures. Outcrossing plant species like *Ficus hirta* Vahl [26], *Alnus glutinosa* f. aurea (Verschaff.) H.J.P.Winkl. [27] and *Camellia sinensis* (L.) Kuntze [28] also reported high heterozygosity values compared to *Atalantia* sp. Populations in heavily disturbed habitats, such as PL and MA for *A. ceylanica*, showed particularly low *H_o_*, whereas isolated populations like MI for *A. buxifolia* maintained higher heterozygosity, suggesting that intact habitats may promote cross-pollination and maintain the genetic variability. These findings underline the importance of conservation strategies to preserve genetic diversity in fragmented or endemic *Atalantia* populations. Similar patterns have been observed in the studies of [13,29,30].

AMOVA indicated that in *A. buxifolia*, 32% of genetic variation occurred among populations and 68% within individuals, while in *A. ceylanica*, 18% was among populations and 82% within populations. Corresponding *F_ST_* values were 0.356 and 0.204, showing significant genetic differentiation. This pattern of higher within-population variation is common in outcrossing Rutaceae; similar trends have been observed in Indonesian tangerine and mandarin accessions [31], wild *Zanthoxylum* species [32], Mediterranean *Citrus* cultivars [33], and northeast Indian Khasi mandarins (*F_ST_* = 0.29) [34]. The elevated within-individual diversity in *Atalantia* likely reflects self-incompatibility and an outcrossing mating system [35], while high observed heterozygosity and a statistically insignificant *F_IS_* value may result from small population sizes rather than extensive gene flow [36,37]. Although heterozygote excess may partly arise from technical factors such as null alleles or sampling effects, null allele frequencies were not explicitly estimated in the present study, and their potential influence cannot be excluded. In addition, recent admixture among lineages or demographic processes such as population recovery after bottlenecks may contribute to heterozygote excess. These alternative explanations suggest that the observed negative *F*_IS_ values likely reflect a combination of biological and technical factors and require further investigation using larger sample sizes [37]. In their study of *Atalantia* sp. in Western Ghats, [2] also observed high within-population genetic variation, but they attributed it to the hybridization events between two species.

The PCoA results supported the AMOVA findings, showing clear population clustering according to geographic regions. In *A. buxifolia*, three distinct clusters were observed along the PC1, with populations from Pingtung and Kaohsiung mostly separated. The Shoushan (SH) population was further distinct along PC2, suggesting it is relatively isolated, likely due to its elevation and surrounding urban environment acting as barriers to gene flow. Even small geographic features can influence plant population structure, as seen in *Titanotrichum oldhamii* (Hemsl.) Soler. and *Salix kusanoi* (Hayata) C.K.Schneid., where isolation shaped genetic diversity [38,39]. For *A. ceylanica*, PCoA revealed distinct clustering by geographic region, separating the MO population along the first axis, similarly to the STRUCTURE results. However, no separation among the other three populations was observed corresponding to their geographical proximity [40].

STRUCTURE analysis of *A. buxifolia* using the ΔK method [41] indicated K = 2 as the most likely number of clusters, broadly separating populations from Kaohsiung and Pingtung. However, visual inspection of bar plots suggested that K = 3 better captured biologically meaningful substructure by further dividing the Kaohsiung populations and isolating the Shoushan (SH) population, which showed limited admixture. For *A. ceylanica*, ΔK = 2 also identified two main genetic clusters, grouping PL, AT, and MA together while separating the Morawaka (MO) population, with minimal admixture. Visual inspection supported K = 3 as a more informative solution, highlighting gene flow among PL, AT, and MA while emphasizing the genetic distinctiveness of MO, likely reflecting its geographic and topographic isolation and environmental barriers such as rugged terrain and landslides [42]. These STRUCTURE patterns were consistent with DAPC results, which recovered three genetic clusters in *A. buxifolia* (separating SH) and two in *A. ceylanica* (separating MO). Similar clustering patterns have been reported in related studies [43,44]. Overall, the observed genetic structure aligns with geographic differentiation, although limited SSR marker polymorphism and uneven sample sizes may influence cluster resolution. As ΔK primarily detects upper hierarchical structure and may underestimate the true number of genetic groups, K = 3 was considered biologically meaningful because it revealed additional substructure supported by DAPC, PCoA, and *F_ST_* analyses [41].

For further verification, we conducted pairwise *F_ST_* testing for both species. In *A. buxifolia*, the SH population showed consistently high pairwise *F*_ST_ values with nearly all other populations, indicating strong genetic divergence and limited contemporary gene flow. Its differentiation from AL, MI, NP, KE, DA, CH, and GU (*F_ST_* > 0.35 in most cases) suggests long-term isolation, likely driven by restricted dispersal and local demographic history, compared with the more interconnected CH–GU–DA–KE cluster, which is located in Pingtung. Population SH appears genetically distinct, contributing unique allelic variation to the species. Similar patterns of strong peripheral differentiation have been observed in other plant species with fragmented habitats [30]. Similarly, in *A. ceylanica*, the MO population was highly differentiated from all others, with *F_ST_* values ranging from 0.312 to 0.403, suggesting strong genetic isolation. This pattern indicates the presence of a cluster of closely related populations (PL–AT–MA) and a single genetically distinct and potentially isolated population (MO) [45].

Barrier analysis using the Monmonier algorithm identified a significant genetic discontinuity separating the SH population of *A. buxifolia* from other Kaohsiung populations, corresponding to the Shoushan mountain range and indicating restricted gene flow [46]. In contrast, Pingtung populations showed no significant barriers, suggesting higher connectivity. The SH population exhibited high genetic distances (0.680–0.685) from neighboring sites, exceeding the threshold for barrier significance and suggesting strong isolation [47]. For *A. ceylanica*, the analysis did not detect clear barriers, so it was not included in the final visualization.

As the above analyses indicated that all *A. buxifolia* populations located in the Pingtung area exhibited low genetic differentiation, we merged them into a single composite population (“PI”) for the BayesAss analysis. Most populations showed strong self-recruitment, with PI exhibiting the highest value (95.40%). The SH population also demonstrated a high self-recruitment rate (94.40%). Strong migration signals were detected between the AL and LO populations (0.18), as well as between the MI and LO populations (0.244), suggesting local connectivity among these populations [48].

Similarly, *A. ceylanica* populations exhibited high self-recruitment, particularly in the geographically isolated MO population (93.80%), reflecting strong genetic isolation. The PL, AT, and MA populations also maintained substantial self-recruitment (68–69%), supporting restricted dispersal. Notably, directional gene flow was observed from MA to PL and AT, with migration rates of 27–29%, whereas most other pairwise migration rates remained low (<3%), consistent with limited overall connectivity.

The results suggest moderate bidirectional gene flow among PL, AT, and MA, with particularly strong exchanges from PL to MA and MA to AT. In contrast, the MO population exhibited high self-recruitment and limited immigration, indicating a relatively isolated genetic structure. These patterns are like those observed in other isolated endemic species, such as *Salix kusanoi* in the island of Taiwan [39]. Because BayesAss requires large sample sizes per population and highly polymorphic microsatellite markers to accurately estimate recent migration rates, the results obtained for *A. ceylanica* should be considered exploratory. Because sample sizes were below the recommended thresholds and several loci were monomorphic, BayesAss estimates should be regarded as preliminary and interpreted with caution. Additional sampling and more variable loci will be necessary to draw firm conclusions about recent migration events among populations [49].

*Atalantia buxifolia* is relatively well distributed across its native range, but its populations may be influenced by ecological and anthropogenic factors. Seed dispersal is primarily mediated by frugivorous birds, which can promote gene flow; however, the degree to which urbanization or habitat disturbance affects dispersal in this species remains uncertain [50]. The Shoushan Mountain (SH) population occurs in an area with a history of military activity and land modification, including limestone mining and increased recreational use [51,52]. Although these activities could potentially affect local habitat structure or population connectivity, their specific demographic impacts on *A. buxifolia* have not been formally assessed. Other Kaohsiung populations (AL, LO, MI) occur in more urbanized and fragmented settings, but whether this fragmentation reduces gene flow is also unclear. In contrast, most Pingtung populations inhabit less disturbed environments, except for the NP population, which consists of only two planted individuals.

Effective conservation of *A. buxifolia* requires a combination of in situ and ex situ strategies. Protecting natural habitats, particularly in Shoushan Mountain and northern Kaohsiung, is essential, and establishing habitat corridors or green bridges could support frugivorous birds and maintain connectivity [53,54]. Integrating the species into urban greening or roadside planting, with different sources to avoid genetic bottlenecks, can involve local communities while supporting conservation [55].

Similarly, *A. ceylanica* faces a high risk of habitat loss. Portions of the PL population have already been destroyed by anthropogenic activities, emphasizing the need for urgent habitat protection. Although some *A. ceylanica* populations (PL, AT, MA) remain relatively undisturbed, AT and MA are near human settlements, increasing their vulnerability, while the MO population, despite being in a rural area, faces anthropogenic disturbances and limited connectivity. Both *A. buxifolia* and *A. ceylanica* are affected by habitat fragmentation, urbanization, and human-mediated pressures, such as pruning or removal due to their thorny morphology. The species’ reliance on frugivorous birds for seed dispersal further highlight the importance of maintaining habitat connectivity. Given their ecological and genetic value, conservation efforts should combine in situ measures protecting natural habitats, establishing ecological corridors, and creating buffer zones with ex situ strategies like propagation in botanical gardens and seed banks. Engaging local communities, promoting sustainable harvesting, and raising awareness are essential, particularly for *A. ceylanica*, which is threatened by habitat loss and overexploitation. Currently, *A. ceylanica* is listed as Data Deficient and *A. buxifolia* as Least Concern [56], highlighting the need for improved conservation prioritization and systematic ecological monitoring. The detection of genetic barriers and limited interpopulation gene flow has important implications for the conservation management of *Atalantia* populations. In situ conservation should prioritize the protection of multiple genetically differentiated populations, particularly isolated lineages such as the Shoushan population in *A. buxifolia*, which may represent unique genetic units. Given the restricted gene flow detected in both species, populations may need to be managed as partially independent conservation units, and translocations or assisted gene flow should be carefully evaluated with the maintenance of overall genetic diversity. Ex situ conservation programs should sample individuals from multiple populations to preserve overall genetic diversity and avoid genetic homogenization.

A limitation of the present study is that sampling was restricted to island populations of *A. buxifolia* in the island of Taiwan and *A. ceylanica* in Sri Lanka, excluding mainland populations from Southeast Asia and southern India, respectively. As island populations often represent peripheral or isolated parts of a species’ range, levels of genetic diversity and population differentiation observed here may not fully reflect patterns across the entire species’ distribution. In particular, reduced diversity or elevated differentiation may partly result from geographic isolation, founder effects, or restricted gene flow characteristic of insular populations. Therefore, the estimates of polymorphism, population structure, and *F_ST_* reported in this study should be interpreted as representative of island lineages rather than of the complete species gene pools. Nevertheless, these data provide a first comparative insight into genetic variation in two geographically disjunct island populations and establish a baseline for future studies incorporating mainland populations to more fully resolve evolutionary history and conservation patterns within *Atalantia*.

## 4. Materials and Methods

### 4.1. Study Species and Sampling

**Figure 5:**
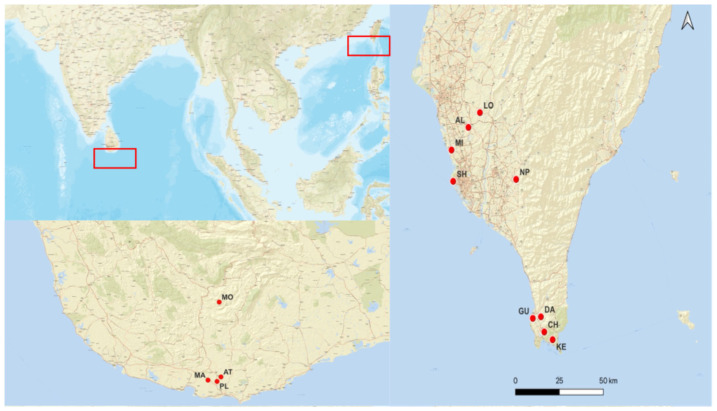
Geographic distribution of sampled populations of *A. buxifolia* in island of Taiwan and *A. ceylanica* in Sri Lanka. Population codes and symbols correspond to locations shown on the map.

To assess genetic diversity and population structure, we collected 58 individuals from four populations of *Atalantia ceylanica* and 74 individuals from nine populations of *Atalantia buxifolia* across Southern Sri Lanka and the southern part of the island of Taiwan (Figure 5 and Table 4). Sampling was restricted to island populations in the island of Taiwan (*A. buxifolia*) and Sri Lanka (*A. ceylanica*) due to the availability of field material and logistical limitations, with the aim of focusing on narrow lineages at the margins of the species’ distributions. Fresh leaves were collected in the field and dried in silica gel prior to DNA extraction. In total, we collected samples of eight wild populations and from a cultivated orchard (NP population).

### 4.2. Extraction of Genomic DNA and Microsatellite PCR Reaction

Genomic DNA was extracted from leaf tissues using the RBC Real Genomics™ Box YGP 100 Kit DNA (RBC Bioscience, New Taipei City, Taiwan) according to the manufacturer’s protocol. DNA quality and concentration were assessed using a Nano-300 Micro-spectrophotometer (Allsheng, Hangzhou, China). Samples were diluted to 5 ng μL^−1^ and stored at −20 °C until further use. A total of 34 microsatellite primer pairs previously developed for *Citrus* [20] were screened to identify transferable and polymorphic loci suitable for *Atalantia*. Gradient PCR with annealing temperatures between 50 °C and 60 °C was conducted to optimize primer performance. Amplification success was evaluated on 1.5% agarose gels. Of the 34 primers tested, 21 produced clear and reproducible bands and were selected for further analyses (Table S1). PCR amplifications were performed on representative samples from each species using optimized conditions. Each 20 μL reaction contained 14.3 μL PCR-grade water, 2 μL dNTP mix, 2 μL 10× buffer, 0.2 μL BSA, 0.5 μL Taq DNA polymerase, and 1 μL genomic DNA. The thermal cycling program consisted of an initial denaturation at 94 °C for 2 min; 40 cycles of 94 °C for 45 s, 58 °C for 1 min, and 72 °C for 1 min; followed by a final extension at 72 °C for 47 min. PCR products were tested on 1.5% agarose gels, and fragment analysis was carried out using an Agilent Fragment Analyzer (Agilent Technologies, Santa Clara, CA, USA). The resulting microsatellite data were used to evaluate genetic diversity and population structure within and among *Atalantia* populations.

### 4.3. Data Analysis

Genetic diversity parameters, including the number of observed alleles (*A*), number of effective alleles (*A_e_*), observed heterozygosity (*H_o_*), and expected heterozygosity (*H_e_*), were calculated for each locus using the R packages adegenet [17], poppr [57,58], and pegas [59]. Deviations from Hardy–Weinberg equilibrium (HWE) were tested for all loci in each population using GenAlEx v6.5 [60]. Overall inbreeding (*F_IT_*), degree of inbreeding (*F_IS_*), and population differentiation (*F_ST_*) were also computed in GenAlEx to assess genetic structure. Analysis of molecular variance (AMOVA) was conducted in GenAlEx v6.5 [60] to partition genetic variation at two hierarchical levels among and within populations.

To visualize patterns of genetic clustering, Principal Coordinate Analysis was performed using GenAlEx v6.5 [60]. The first two Principal Component (PC) axes, explaining the highest proportion of genetic variance, were used to interpret population structure and differentiation. Population structure was further assessed using STRUCTURE v2.3.4 [61] under a Bayesian clustering framework with correlated allele frequencies and an admixture model. Each run consisted of 5 × 10^6^ iterations following a burn-in period of 3 × 10^6^, and analyses were repeated 15 times with different random seeds to ensure convergence. The most likely number of genetic clusters (K) was determined using the ΔK method of [41], implemented in Structure Selector [62]. To visualize genetic relationships among populations, Discriminant Analysis of Principal Coordinates (DAPC) was conducted with the adegenet package in R [17]. The first two Linear Discriminants for *A. buxifolia* and first three Linear Discriminants for *A. ceylanica*, which accounted for the highest proportion of genetic variance and were used to deduce patterns of clustering.

To detect genetic boundaries, Monmonier’s algorithm implemented in adegenet package in R [17] was applied to georeferenced *Atalantia buxifolia* populations using a Delaunay triangulation to identify regions of highest genetic differentiation. Genetic differentiation of populations was assessed by pairwise *F_ST_* computed in GenAlEx v6.5 [60] and visualized in R studio. Finally, recent migration rates among populations were estimated using BayesAss v3.0.5 [49]. The Markov Chain Monte Carlo (MCMC) analysis consisted of 1,000,000 iterations, with the first 10,000 runs discarded as burn-in. As in *A. ceylanica*, the markers exhibited low polymorphism and the sampling sizes are uneven, and the migration estimates might introduce uncertainty. So, it is only used as preliminary findings. The artificially established NP population in *A. buxifolia* was excluded in barrier analysis and migration analysis to avoid bias in estimating natural gene flow.

## 5. Conclusions

This study demonstrates clear genetic structuring and moderate to high genetic diversity in *Atalantia buxifolia* and *Atalantia ceylanica* populations from the island of Taiwan and Sri Lanka, respectively. Microsatellite analyses revealed substantial variation within populations and differentiation among them, shaped by geographic isolation, habitat fragmentation, and limited gene flow. Clustering and multivariate analyses confirmed regional genetic structuring, providing a valuable baseline for conservation planning. Wild *Atalantia* populations represent important reservoirs of genetic diversity, which could support future *Citrus* breeding for stress tolerance and disease resistance. Conserving these species requires a combination of in situ habitat protection, ex situ propagation, and community engagement to ensure long-term survival, particularly for populations under anthropogenic pressure or near human settlements.

## Figures and Tables

**Table 1:** Analysis of molecular variance (AMOVA) for nine populations of *Atalantia buxifolia* based on 21 microsatellite loci.

Source of Variation	df	Sum of Squares	Mean Squares	Estimated Variance	Percentage of Variation	Fixation Index
Among populations	8	250.616	31.327	1.847	32%	*F_ST_* = 0.356 *
Within individuals	74	294.000	3.973	3.973	68%	*F_IS_* = −0.189
Total	82	544.616		5.820	100%	*F_IT_* = 0.234 *

*: *p* < 0.05; d.f.: degree of freedom.

**Table 2:** Analysis of molecular variance (AMOVA) for four populations of *Atalantia ceylanica* based on 21 microsatellite loci.

Source of Variation	df	Sum of Squares	Mean Squares	Estimated Variance	Percentage of Variation	Fixation Index
Among populations	3	57.725	19.242	0.615	18%	*F_ST_* = 0.204 *
Within individuals	58	168.000	2.897	2.897	82%	*F_IS_* = −0.207
Total	61	225.725		3.511	100%	*F_IT_* = 0.039

*: *p* < 0.05; d.f.: degree of freedom.

**Table 3:** Pairwise migration matrix among populations of *Atalantia*. Values represent posterior mean migration probabilities with standard deviations in parentheses. The first five (SH, AL, LO, MI, PI) belong to *A. buxifolia*, whereas the latter four populations (PL, AT, MA, MO) represent *A. ceylanica*. Migration estimates are shown only within species groups; between-species estimates are not available.

From\To	SH	AL	LO	MI	PI	PL	AT	MA	MO
SH	0.9466 (0.0241)	0.0134 (0.0130)	0.0134 (0.0129)	0.0134 (0.0129)	0.0133 (0.0129)	—	—	—	—
AL	0.0376 (0.0339)	0.7039 (0.0331)	0.1838 (0.0530)	0.0374 (0.0335)	0.0372 (0.0334)	—	—	—	—
LO	0.0245 (0.0216)	0.0187 (0.0179)	0.9199 (0.0341)	0.0183 (0.0174)	0.0186 (0.0181)	—	—	—	—
MI	0.0225 (0.0212)	0.0221 (0.0210)	0.2443 (0.0368)	0.6889 (0.0207)	0.0223 (0.0210)	—	—	—	—
PI	0.0114 (0.0109)	0.0115 (0.0111)	0.0114 (0.0111)	0.0116 (0.0113)	0.9540 (0.0207)	—	—	—	—
PL	—	—	—	—	—	0.6806 (0.0148)	0.0169 (0.0158)	0.2889 (0.0248)	0.0135 (0.0130)
AT	—	—	—	—	—	0.0243 (0.0226)	0.6912 (0.0229)	0.2608 (0.0359)	0.0238 (0.0222)
MA	—	—	—	—	—	0.0230 (0.0219)	0.2735 (0.0330)	0.6852 (0.0203)	0.0183 (0.0174)
MO	—	—	—	—	—	0.0208 (0.0196)	0.0208 (0.0196)	0.0208 (0.0195)	0.9375 (0.0316)

**Table 4:** Sample collection from the island of Taiwan and Sri Lanka.

Species	Populations	Symbols	GPS Coordinates	No. of Individuals
*A. buxifolia*	Shoushan	SH	22.63528, 120.26139	20
	Alian	AL	22.86486, 120.34594	4
	Longchi	LO	22.92764, 120.40936	13
	Mituo	MI	22.76875, 120.25228	10
	Neipu	NP	22.64350, 120.60980	2
	Kenting	KE	21.95912, 120.81186	4
	Dashanmu Mt.	DA	22.05720, 120.74711	5
	Chiniuiling	CH	21.99246, 120.76573	11
	Guishan	GU	22.05056, 120.70221	5
*A. ceylanica*	Palatuwa	PL	5.98639, 80.52028	21
	Attudawa	AT	5.99972, 80.53194	10
	Malimboda	MA	5.99028, 80.49333	15
	Morawaka	MO	6.22250, 80.52667	12
Total				132

## Data Availability

The original contributions in the study are included in the Supplementary Material as raw data sheets. Further inquiries can be directed to the corresponding author.

## Supplementary Materials

The following supporting information can be downloaded at https://www.mdpi.com/article/10.3390/plants15040570/s1. Table S1: Specific primer sequences, basic motif units, expected fragment sizes and original references for 34 microsatellite loci. Table S2: Raw data of 74 samples from *Atalantia buxifolia* populations in the island of Taiwan based on amplicon sizes using 21 SSR loci. Table S3: Raw data of 58 samples from *Atalantia ceylanica* populations in Sri Lanka based on amplicon sizes using 21 SSR loci. Table S4: STRUCTURE results for K = 1–10 and ΔK statistics calculated using the Evanno method [41] for *Atalantia buxifolia*. Table S5: STRUCTURE results for K = 1–8 and ΔK statistics calculated using the Evanno method [41] for *Atalantia Ceylanic.* Figure S1: Results of STRUCTURE model selection using the Evanno method for *Atalantia buxifolia*. Figure S2: Results of STRUCTURE model selection using the Evanno method for *Atalantia ceylanica*. Figure S3: DAPC analysis for *Atalantia* sp.
