# Supplementary material for: Comparative Genetic Diversity and Population Structure of Wild Atalantia from Taiwan and Sri Lanka Using SSR Markers
Source: Plants (Basel). 2026 Feb 11;15(4):570. doi: 10.3390/plants15040570 (PMC12944599; doi:10.3390/plants15040570)
Supplement: Supplementary file 1 [file plants-15-00570-s001.zip › plants-4123187-supplementary.pdf]

Table S1: Description of microsatellite loci used in *Atalantia* sp. The primers are from Ji et al., 2012, the original references that the primers were described are also mentioned here.

**Primer code**	**Sequence**	**Repeat motif**	**Allele size**	**Original Reference for the primer**
**Cit_2**	F:TCAAACATCAGACGAAGCAA<br>R:TGAATCTTTTGCCGAATTTTG	(A)18	179-193	Ahmad et al.,2003
**Cit_3**	F:CGTGCAGAGAAACTCAGATCC<br>R: GCTGAAAAAGATTCATTTTGCC	(CA)10	156-169	Ahmad et al.,2003
**Cit_4**	F:CCAAACATCTGCGGATCC<br>R: AGAAGAACCCAGATTCCAAATG	(CT)11	140-148	Ahmad et al.,2003
**Cit_7**	F: GCTCCTCGAATGAGAATGAAATGA<br>R: TGGTTGTGCGAAAATGAAGAGATA		150-280	Tonogbanua et al.,2018
**Cit_8**	F: AAAAATCGAAATCGAGCACCC<br>R: GAAGTAACGGAGAATTCCGATGAG		130-300	Tonogbanua et al.,2018
**Cit_9**	F: TTCACCACAAACGAAGACTCAGAC<br>R:CTGTAATCCACTCGGTAATCCGAC		135-170	Tonogbanua et al.,2018
**Cit_10**	F: CGAAGAAGAATTGAAAGAGCCAGA<br>R: CAACAGATTTGTTACTGGAAGGGG		140-220	Tonogbanua et al.,2018
**Cit_11**	F: TTCATTTGGAACAAAACCCAATTC<br>R: GCTGCTAATCACAGCATCAAGAGA		140-220	Tonogbanua et al.,2018
**Cit_14**	F: CCTCAGCTCTAGCAAAAGCACATT<br>R: AGAGGCTATAGATCGTGGATGCAG		140-220	Tonogbanua et al.,2018
**Cit_15**	F: TTTATTCACCGCTCAAGGACT<br>R: TTAGGGGTGGAAAACATGGA		210-480	Tonogbanua et al.,2018
**Cit_18**	F: ATACGATGCGTGAACTGC<br>R: TACCTTTCTTTCTCCTCTGT		160-200	Tonogbanua et al.,2018
**Cit_19**	F: AAAGGGAAAGCCCTAATCTCA<br>R: CTTCCTCTTGCGGAGTGTTC	AG	120-240	Barkley et al.,2006
**Cit_20**	F: GCTTTCGATCCCTCCACATA<br>R: GATCCCTACAATCCTTGGTCC	CAT	120-260	Barkley et al.,2006

**Cit_23**	F: AATGCTGAAGATAATCCGCG<br>R: TGCCTTGCTCTCCACTCC	AGG	140-180	Barkley et al.,2006
**Cit_24**	F: ACAACCTTCAACAAAACCTAGG<br>R: AAGACTTGGTGCGACAGG	CAC	140-180	Nematollahi et al.,2013
**Cit_25**	F: AACACCCCTTGGAGGGAG<br>R: GCTGTTCACACACACAACCC	(CT)9<br>(CA)5	146-170	Ahmad et al.,2003
**Cit_26**	F: TTATTGTCCCCAATTGTGAGC<br>R: TCCAGATTGAGGGGAAAAAG	(CA)20<br>(TA)5	123-169	Ahmad et al.,2003
**Cit_29**	F: CAGGATGCTTGTTGGTGATG<br>R: ACAGTGGATACAAACATGCTGC	(CT)16	144-154	Ahmad et al.,2003
**Cit_30**	F: TAAATCTCCACTCTGCAAAAGC<br>R: GATAGGAAGCGTCGTAGACCC	CAC	148-163	Barkley et al.,2006
**Cit_31**	F: TATGGTGGCAGATTAACAGCC<br>R: TACTCCGGAGAAAGATTTGGG	(CT)10	161-166	Ahmad et al.,2003
**Cit_34**	F: GCACCTTTTATACCTGACTCGG<br>R: TTCAGCATTTGAGTTGGTTACG	TAA	120-142	Shahzadi et al.,2014

Table S2: Genetic diversity metrics for *Atalantia buxifolia* populations in Taiwan were assessed using 21 microsatellite markers. The analysis included the number of alleles (A), effective number of alleles (*Ae*), observed heterozygosity (*Ho*), and expected heterozygosity (*He*). Hardy-Weinberg equilibrium (HWE) at each locus was evaluated using the chi-square test (χ^2), with loci showing significant deviation (P < 0.05) marked with an asterisk (*).

	**SH**					**AL**					**LO**				
**Locus**	***A***	***Ae***	***Ho***	***He***		***A***	***Ae***	***Ho***	***He***		***A***	***Ae***	***Ho***	***He***	
**Cit_2**	3	1.45	0.45	0.484	***	3	1.75	0.75	0.656	[illegible]	2	1.85	0.846	0.488	**
**Cit_3**	2	1.3	0.3	0.255	ns	1	1	0	0		1	1	0	0	
**Cit_4**	1	1	0	0		1	1	0	0		1	1	0	0	
**Cit_7**	2	1.45	0.45	0.499	ns	2	1.5	0.5	0.5	ns	2	1.77	0.769	0.497	*
**Cit_8**	2	1.3	0.3	0.5	ns	2	1.5	0.5	0.375	ns	2	1.46	0.462	0.355	ns
**Cit_9**	2	1	0	0.095	***	1	1	0	0		1	1	0	0	
**Cit_10**	3	1.75	0.75	0.596	ns	2	1	0	0.375	*	5	1.23	0.231	0.598	ns
**Cit_11**	2	1.1	0.1	0.095	ns	1	1	0	0		2	1.31	0.308	0.426	ns
**Cit_14**	1	1	0	0		2	1.25	0.25	0.219	ns	2	1.08	0.077	0.074	ns
**Cit_15**	2	1	0	0.095	***	2	1.5	0.5	0.5	ns	3	1.77	0.769	0.651	ns
**Cit_18**	2	1.15	0.15	0.399	**	3	1.25	0.25	0.594	ns	3	1.31	0.308	0.328	ns
**Cit_19**	3	1.5	0.5	0.515	*	1	1	0	0		2	1.23	0.231	0.311	ns
**Cit_20**	2	1.15	0.15	0.139	ns	2	2	1	0.5	*	2	1.61	0.615	0.473	ns
**Cit_23**	3	1.25	0.25	0.226	ns	2	1.25	0.25	0.219	ns	2	1.46	0.462	0.473	ns
**Cit_24**	2	2	1	0.5	***	2	2	1	0.5	*	2	1.92	0.923	0.497	**
**Cit_25**	2	2	1	0.5	***	2	1.75	0.75	0.469	ns	2	1.85	0.846	0.488	**
**Cit_26**	1	1	0	0		2	1.75	0.75	0.469	ns	2	2	1	0.5	***
**Cit_29**	1	1	0	0		3	2	1	0.594	ns	3	1.61	0.615	0.663	***
**Cit_30**	2	1.05	0.05	0.049	ns	1	1	0	0		3	1.39	0.385	0.651	ns
**Cit_31**	2	2	1	0.5	***	1	1	0	0		1	1	0	0	
**Cit_34**	2	1.7	0.7	0.455	*	2	2	1	0.5	*	2	1.15	0.154	0.142	ns
mean	2	1.34	0.340	0.281		1.81	1.40	0.405	0.308		2.14	1.43	0.429	0.363	

Genetic diversity metrics for *Atalantia buxifolia* populations in Taiwan were assessed using 21 microsatellite markers. The analysis included the number of alleles (A), effective number of alleles (*Ae*), observed heterozygosity (*Ho*), and expected heterozygosity (*He*). Hardy-Weinberg equilibrium (HWE) at each locus was evaluated using the chi-square test (χ^2), with loci showing significant deviation (P < 0.05) marked with an asterisk (*) (continued).

**Locus**	**MI**					**NP**					**KE**				
	A	***Ae***	***Ho***	***He***		***A***	***Ae***	***Ho***	***He***		***A***	***Ae***	***Ho***	***He***	
**Cit_2**	2	2	1	0.5	***	2	2	1	0.5	ıs	4	1.33	0.333	0.722	ns
**Cit_3**	1	1	0	0		1	1	0	0		1	1	0	0	
**Cit_4**	1	1	0	0		1	1	0	0		1	1	0	0	
**Cit_7**	2	1.7	0.7	0.455	ns	2	1.33	0.333	0.278		2	2	1	0.5	ns
**Cit_8**	2	1.3	0.3	0.255	ns	2	1.33	0.333	0.278		1	1	0	0	
**Cit_9**	1	1	0	0		1	1	0	0		1	1	0	0	
**Cit_10**	4	1.4	0.4	0.655	ns	3	1.67	0.667	0.667	ns	3	2	1	0.611	ns
**Cit_11**	2	1.1	0.1	0.095	ns	1	1	0	0		1	1	0	0	
**Cit_14**	2	1	0	0.32	***	1	1	0	0		1	1	0	0	
**Cit_15**	2	1.9	0.9	0.495	**	2	1.67	0.667	0.444	ns	2	1	0	0.444	ns
**Cit_18**	4	1.5	0.5	0.595	ns	2	1.67	0.667	0.444	ns	1	1	0	0	
**Cit_19**	2	1.2	0.2	0.18	ns	2	1	0	0.444		2	2	1	0.5	ns
**Cit_20**	2	1.1	0.1	0.095	ns	2	1.67	0.667	0.444	ns	2	1.33	0.333	0.278	ns
**Cit_23**	2	1.3	0.3	0.255	ns	1	1	0	0		1	1	0	0	
**Cit_24**	2	1.9	0.9	0.495	**	2	2	1	0.5	ns	2	2	1	0.5	ns
**Cit_25**	2	1.9	0.9	0.495	**	2	1.67	0.667	0.444	ns	1	1	0	0	
**Cit_26**	2	2	1	0.5	***	2	2	1	0.5	ns	4	2	1	0.722	ns
**Cit_29**	5	1.6	0.6	0.775	*	2	1	0	0.444		3	1.33	0.333	0.5	ns
**Cit_30**	4	1.4	0.4	0.65	ns	2	1	0	0.444		2	1.33	0.333	0.278	ns
**Cit_31**	1	1	0	0		1	1	0	0		1	1	0	0	
**Cit_34**	2	1.7	0.7	0.455	ns	2	1.33	0.333	0.278		2	1.33	0.333	0.278	ns
mean	2.24	1.43	0.429	0.363		1.71	1.35	0.349	0.291		1.81	1.32	0.321	0.368	

Genetic diversity metrics for *Atalantia buxifolia* populations in Taiwan were assessed using 21 microsatellite markers. The analysis included the number of alleles (A), effective number of alleles (*Ae*), observed heterozygosity (*Ho*), and expected heterozygosity (*He*). Hardy-Weinberg equilibrium (HWE) at each locus was evaluated using the chi-square test (χ^2), with loci showing significant deviation (P < 0.05) marked with an asterisk (*)(continued).

**Locus**	**DA**					**CH**					**GU**				
	A	***Ae***	***Ho***	***He***		***A***	***Ae***	***Ho***	***He***		***A***	***Ae***	***Ho***	***He***	
**Cit_2**	3	1.6	0.6	0.62	ns	2	2	1	0.5	**	2	2	1	0.5	*
**Cit_3**	1	1	0	0		1	1	0	0		1	1	0	0	
**Cit_4**	1	1	0	0		1	1	0	0		1	1	0	0	
**Cit_7**	2	1.4	0.4	0.32	ns	2	1.36	0.364	0.298	ns	2	1.2	0.2	0.18	ns
**Cit_8**	2	1.4	0.4	0.32	ns	1	1	0	0		2	1.2	0.2	0.18	ns
**Cit_9**	1	1	0	0		1	1	0	0		1	1	0	0	
**Cit_10**	4	2	1	0.7	ns	5	1.82	0.818	0.715	ns	5	1.8	0.8	0.68	ns
**Cit_11**	1	1	0	0		1	1	0	0		1	1	0	0	
**Cit_14**	2	1.2	0.2	0.18	ns	2	1.46	0.455	0.351	ns	2	1.4	0.4	0.32	ns
**Cit_15**	1	1	0	0		1	1	0	0		1	1	0	0	
**Cit_18**	1	1	0	0		2	1.36	0.364	0.298	ns	1	1	0	0	
**Cit_19**	3	1.8	0.8	0.58	ns	3	1.73	0.727	0.616	ns	3	1.8	0.8	0.54	ns
**Cit_20**	2	1.8	0.8	0.48	ns	2	1.46	0.455	0.483	ns	2	2	1	0.5	*
**Cit_23**	1	1	0	0		1	1	0	0		1	1	0	0	
**Cit_24**	2	2	1	0.5	*	2	2	1	0.5	***	2	2	1	0.5	*
**Cit_25**	1	1	0	0		1	1	0	0		1	1	0	0	
**Cit_26**	4	1.2	0.2	0.74	ns	4	1.64	0.636	0.682	ns	3	1.6	0.6	0.46	ns
**Cit_29**	6	2	1	0.8	ns	4	1.82	0.818	0.694	ns	3	1.4	0.4	0.54	ns
**Cit_30**	3	1.8	0.8	0.58	ns	2	1.73	0.727	0.496	ns	3	1.6	0.6	0.62	ns
**Cit_31**	1	1	0	0		1	1	0	0		1	1	0	0	
**Cit_34**	2	1.2	0.2	0.18	ns	2	1.64	0.636	0.434	ns	2	1.4	0.4	0.32	ns
mean	2.10	1.35	0.352	0.286		1.95	1.38	0.381	0.289		1.90	1.35	0.352	0.254	

Table S3: Genetic diversity metrics for *Atalantia ceylanica* populations in Sri Lanka were assessed using 21 microsatellite markers. The analysis included the number of alleles (A), effective number of alleles (*Ae*), observed heterozygosity (*Ho*), and expected heterozygosity (*He*). Hardy-Weinberg equilibrium (HWE) at each locus was evaluated using the chi-square test (χ^2), with loci showing significant deviation ($P < 0.05$) marked with an asterisk (*).

Locus	PL					AT					MA				
	A	*Ae*	*Ho*	*He*		*A*	*Ae*	*Ho*	*He*		*A*	*Ae*	*Ho*	*He*	
**Cit_2**	2	1	0	0.091	***	2	1	0	0.42	**	3	1.13	0.133	0.424	*
**Cit_3**	2	1.57	0.571	0.472	ns	2	1.1	0.1	0.375	*	2	1.07	0.067	0.064	
**Cit_4**	1	1	0	0		1	1	0	0		1	1	0	0	
**Cit_7**	2	1.33	0.333	0.278	ns	2	1.4	0.4	0.32	ns	2	1.07	0.067	0.18	ns
**Cit_8**	1	1	0	0		1	1	0	0		1	1	0	0	ns
**Cit_9**	3	1.09	0.095	0.251	**	2	1.2	0.2	0.32	ns	2	1.13	0.133	0.124	
**Cit_10**	2	1.95	0.952	0.499	***	2	1.9	0.9	0.495	**	2	2	1	0.5	ns
**Cit_11**	2	1.05	0.048	0.046	ns	1	1	0	0		2	1.13	0.133	0.124	
**Cit_14**	1	1	0	0		1	1	0	0		1	1	0	0	ns
**Cit_15**	1	1	0	0		1	1	0	0		1	1	0	0	
**Cit_18**	4	1.33	0.333	0.661	**	4	1.7	0.7	0.7	ns	4	1.53	0.533	0.7	
**Cit_19**	2	1.29	0.286	0.408	ns	2	1.7	0.7	0.455	ns	2	1.53	0.533	0.498	ns
**Cit_20**	2	1.95	0.952	0.499	***	2	1.4	0.4	0.42	ns	1	1	0	0	*
**Cit_23**	1	1	0	0		1	1	0	0		1	1	0	0	
**Cit_24**	4	1.95	0.952	0.748	***	4	2	1	0.59	***	4	2	1	0.74	*
**Cit_25**	1	1	0	0		1	1	0	0		1	1	0	0	
**Cit_26**	3	1.38	0.381	0.608	***	3	1.9	0.9	0.585	***	3	1.8	0.8	0.604	ns
**Cit_29**	2	2	1	0.5	***	2	2	1	0.5	**	2	1.4	0.4	0.32	ns
**Cit_30**	1	1	0	0		1	1	0	0		1	1	0	0	ns
**Cit_31**	3	1.71	0.714	0.489	ns	2	1.8	0.8	0.48	*	2	1.67	0.667	0.444	
**Cit_34**	1	1	0	0		1	1	0	0		1	1	0	0	ns
mean	1.95	1.31	0.315	0.264		1.81	1.34	0.338	0.270		1.86	1.26	0.260	0.225	

Genetic diversity metrics for *Atalantia ceylanica* populations in Sri Lanka were assessed using 21 microsatellite markers. The analysis included the number of alleles (A), effective number of alleles (*Ae*), observed heterozygosity (*Ho*), and expected heterozygosity (*He*). Hardy-Weinberg equilibrium (HWE) at each locus was evaluated using the chi-square test (χ^2), with loci showing significant deviation (P < 0.05) marked with an asterisk (*) (continued).

**Locus**	**MO**				
	A	***Ae***	***Ho***	***He***	
**Cit_2**	1	1	0	0	
**Cit_3**	2	1.25	0.25	0.219	ns
**Cit_4**	1	1	0	0	
**Cit_7**	1	1	0	0	
**Cit_8**	1	1	0	0	
**Cit_9**	1	1	0	0	
**Cit_10**	2	2	1	0.5	***
**Cit_11**	1	1	0	0	
**Cit_14**	1	1	0	0	
**Cit_15**	1	1	0	0	
**Cit_18**	2	1.08	0.083	0.08	ns
**Cit_19**	2	1	0	0.153	***
**Cit_20**	1	1	0	0	
**Cit_23**	1	1	0	0	
**Cit_24**	2	2	1	0.5	***
**Cit_25**	1	1	0	0	
**Cit_26**	1	1	0	0	
**Cit_29**	2	1.33	0.333	0.278	ns
**Cit_30**	1	1	0	0	
**Cit_31**	2	2	1	0.5	***
**Cit_34**	1	1	0	0	
mean	1.33	1.17	0.175	0.264	

Table S4: STRUCTURE results for K = 1–10 and ΔK statistics calculated using the Evanno method (Evanno et al., 2005) for *Atalantia buxifolia*.

# K	Reps	Mean LnP(K)	Stdev LnP(K)	Ln'(K)	Ln''(K)	Delta K
1	15	-2636.6333	0.24103	NA	NA	NA
2	15	-2045.7867	0.11255	590.84667	345.29333	3068.01178
3	15	-1800.2333	0.09759	245.55333	219.94667	2253.78266
4	15	-1774.6267	0.46209	25.60667	15.81333	34.2216
5	15	-1764.8333	1.90175	9.79333	19.82667	10.42547
6	15	-1774.8667	7.52251	-10.03333	36.87333	4.90174
7	15	-1821.7733	6.98503	-46.90667	49.00667	7.01595
8	15	-1819.6733	16.20134	2.1	6.12	0.37775
9	15	-1823.6933	15.47599	-4.02	4.68667	0.30283
10	1	-1832.4	0	-8.70667	NA	NA

Table S5: STRUCTURE results for K = 1–8 and ΔK statistics calculated using the Evanno method (Evanno et al., 2005) for *Atalantia ceylanica*.

# K	Reps	Mean LnP(K)	Stdev LnP(K)	Ln'(K)	Ln''(K)	Delta K
1	15	-1108.04	0.18822	NA	NA	NA
2	15	-959.43333	0.53807	148.60667	117.31333	218.02447
3	15	-928.14	0.29713	31.29333	59.22	199.30729
4	15	-956.06667	13.28929	-27.92667	78.10667	5.87741
5	15	-1062.1	22.21354	-106.03333	34.49333	1.55281
6	15	-1133.64	21.67236	-71.54	69.12667	3.18962
7	15	-1136.0533	17.7223	-2.41333	15.51667	0.87554
8	14	-1122.95	14.77538	13.10333	NA	NA

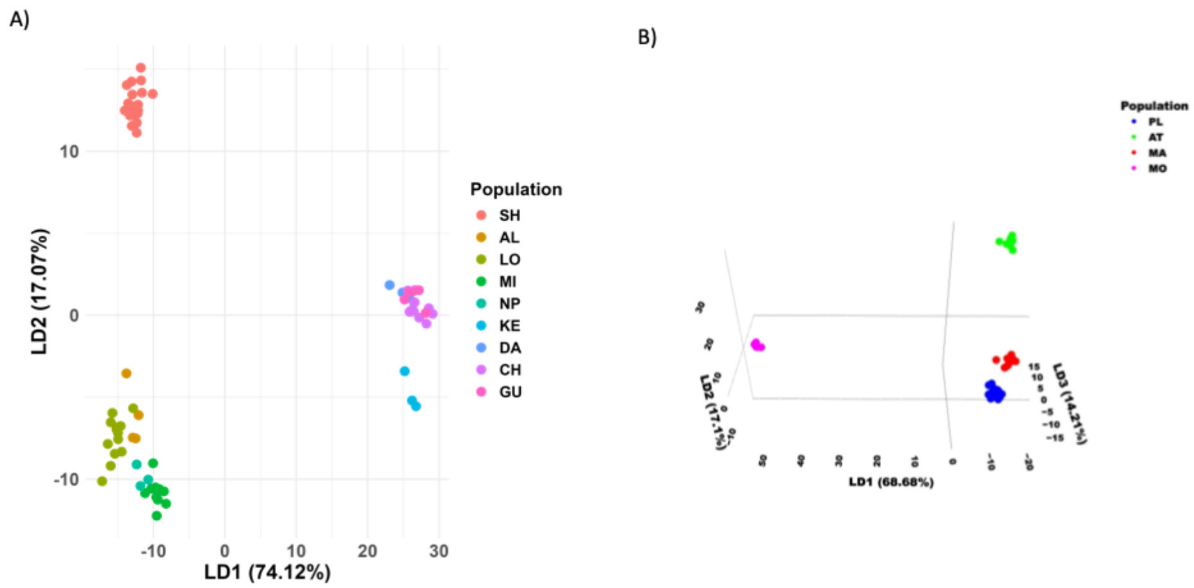

Figure S1: DAPC Analysis of A) *A. buxifolia*, LD1 and LD2 accounts for 91.19% total variance, B*) A. ceylanica*, LD1 – LD3 accounts for 99.99% of total variance.

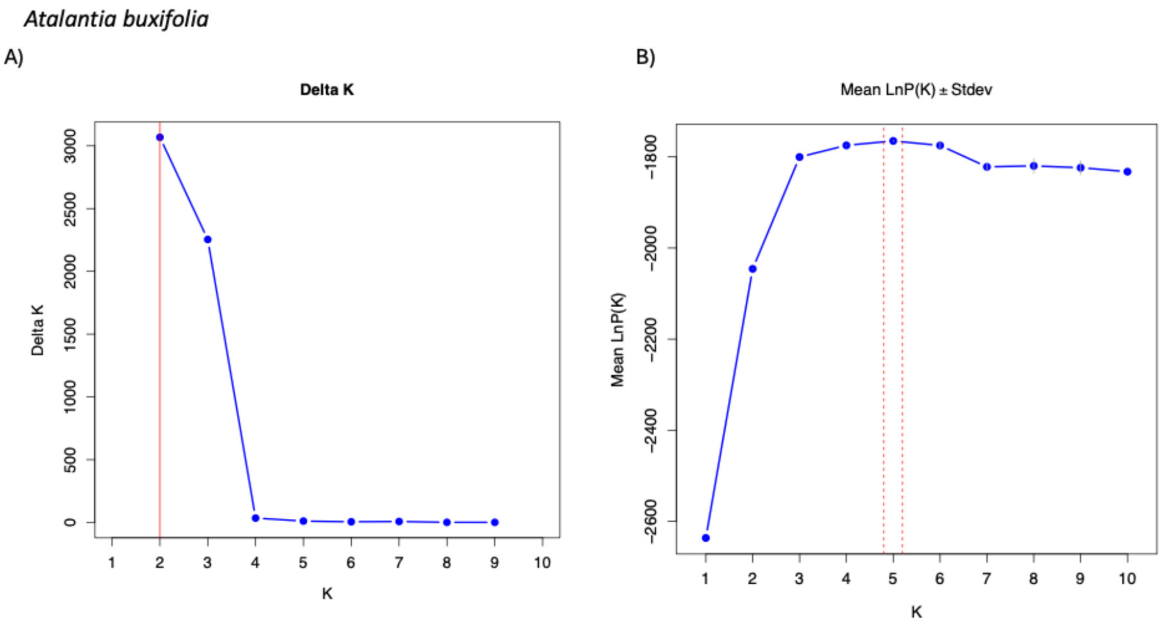

Figure S2: Results of STRUCTURE model selection using the Evanno method for *A. buxifolia*. (a) ΔK plot showing the rate of change in LnP(K), used to infer the optimal number of clusters. (b) Plot of Mean LnP(K) versus K, with standard deviation across replicates, illustrating the likelihood plateau and supporting model selection.

Atalantia ceylanica

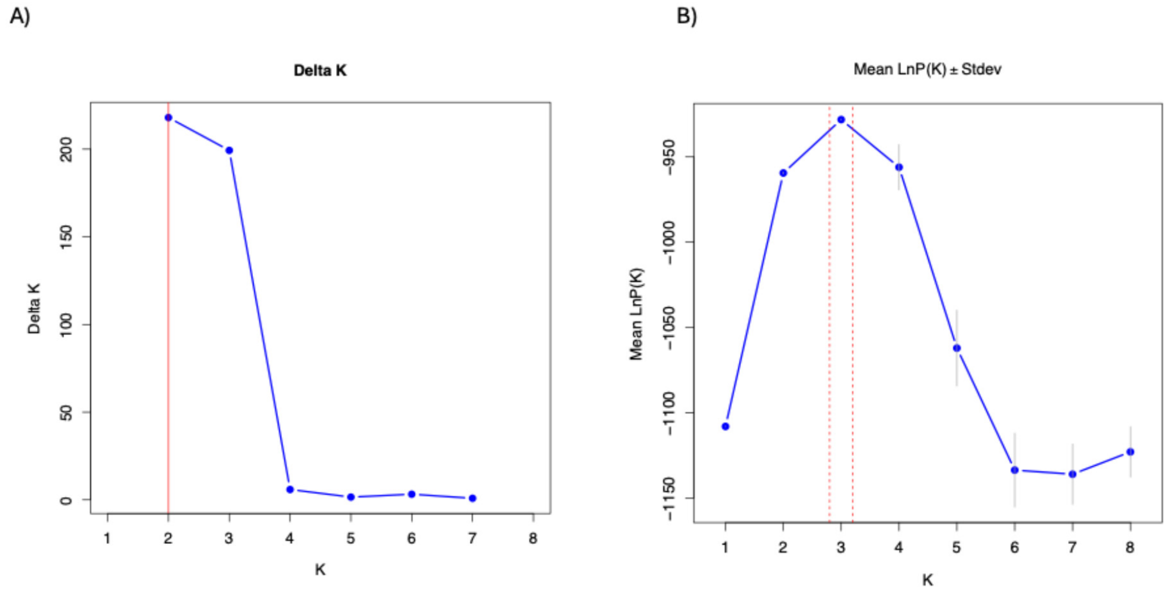

Supplementary Figure S3: Results of STRUCTURE model selection using the Evanno method for *A. ceylanica.*
(a) ΔK plot showing the rate of change in LnP(K), used to infer the optimal number of clusters.
(b) Plot of Mean LnP(K) versus K, with standard deviation across replicates, illustrating the likelihood plateau and supporting model selection.
